# Classification of LED Packages for Quality Control by Discriminant Analysis, Neural Network and Decision Tree

**DOI:** 10.3390/mi15040457

**Published:** 2024-03-28

**Authors:** Heesoo Shim, Sun Kyoung Kim

**Affiliations:** Department of Mechanical System Design Engineering, Seoul National University of Science and Technology, Seoul 01811, Republic of Korea

**Keywords:** machine learning, discriminant analysis, neural network, decision tree, LED

## Abstract

This study investigates supervised learning to improve LED classification. A hardware system for testing was built. The data for learning were acquired and then analyzed to show their characteristics. An LED was tested, and the results were categorized into three defective LED groups and one normal LED group. Before classification, electrical and optical data were examined to identify their characteristics. To find out the best way for quality control, an ensemble of methods was used. First, the discriminant analysis using the validation data achieved a 77.9% true positive rate for normal products, inadequate for quality control. Second, neural network-based learning boosted this rate to 97.8%, but the 2.2% false negative rate remained problematic. Finally, a binary decision tree was constructed, achieving a 99.4% true positive rate from just 14 splits, proving highly effective in product classification. The training time was measured as 8.1, 18.2 and 8.2 s for discriminant analysis, neural network and decision tree, respectively. This work has found the binary decision tree is advantageous considering both learning and classification efficiencies.

## 1. Introduction

In recent years, in conjunction with the growth of the LED (light emitting diode) market for various applications, there has been an expansion into the market for LED lighting [1,2,3,4,5,6]. Consequently, the inspection of LED packages has become a pivotal concern within manufacturing processes [7]. The demands for increased speed and enhanced reliability persist in these inspection procedures. As high-power LED packages continue to be developed, various LED types cater to specific applications. This phase aligns with the preparations for mass producing high-efficiency LED products designed for illumination. As production scales, the necessity for deploying inspection equipment becomes increasingly vital, representing a pivotal moment for advancing reliability measurement tools, enhancing quality standards, and ensuring the objectivity of product quality validation.

There are several advanced LED packages such as flip-chip and wafer-level packages, but SMD (surface mount device) LEDs are still popular for a variety of applications. They have achieved a high-speed processing rate of approximately 20,000 units per hour (UPH). Packaged LEDs should go through inspection before shipping. To further improve speed and reliability, there is a pressing need for enhancements in inspection.

A decade ago, Perng et al. introduced an automated method for inspecting SMD LEDs using machine vision [8]. In a similar vein, Kuo et al. achieved an impressive recognition rate of 97.83% by first extracting features from images through clustering and then training a neural network [9]. More recently, Lin et al. introduced a method for detecting defective LEDs using convolutional neural networks (CNNs) from images of operational LEDs [10]. These techniques offer valuable approaches, streamlining the inspection process and extracting rich information from images. However, it is important to note that real-time image processing demands substantial computational power [11], leading to the continued reliance on classical optical and electrical measurement methods in actual mass production. While these methods alone may not suffice to ensure reliability, they can be complemented by other approaches that facilitate real-time measurements [12].

Smart manufacturing heavily relies on intelligent algorithms for decisions [13]. Recently, data-based quality management techniques have been applied in various cases, and their utility continues to rise. Among these techniques, machine learning is the most popular approach in fields such as design automation [14], semiconductor analysis [15] and modeling [16]. Especially for classifications, machine learning can be a reliable and scalable solution. The classification methods by machine learning have been implemented and tested for recycling [17], precision machining [18], welding [19,20], tools [21], bearing diagnostics [22], consumer parts [23], additive manufacturing [24], human action in manufacturing [25] and polymer processing [26].

In practice, quality controls of SMD LEDs are still conducted using rule-based expert systems. This prevents scale-up and automation of system building. To enhance efficiency, this study specifically examines three key algorithms: discriminant analysis (DA), a well-established statistical technique with a long history of application in data mining; neural networks (NNs), the predominant choice for artificial intelligence-based decision-making; and decision trees (DTs), known for their ability to perform systematic classification through learning. These methods have been applied to classification in manufacturing for automation and smart processes [27,28].

A dedicated automated testing apparatus, custom-tailored for the examination of SMD LEDs for lighting, has been developed for integration into the inspection line, in alignment with the methodology devised in this study. The SMD package in this work includes two LED devices. The package is considered normal when both the LEDs are bright as designed. However, some packages can be fully dark, evenly dimmed, or partly dark. Each defective LED should be sorted into its respective category, while normal ones are classified accordingly. After inspection, the defective LEDs are either repaired, repurposed, or discarded.

Datasets for training and validation were collected to create a robust classification system for the inspection process. Before learning, the data were scrutinized in detail to understand the characteristics of the measurements. Once the classification systems based on the aforementioned three methods were learned on the training dataset, their performance was evaluated on a separate validation dataset.

## 2. Methods

### 2.1. Experimental System

**Figure 1:**
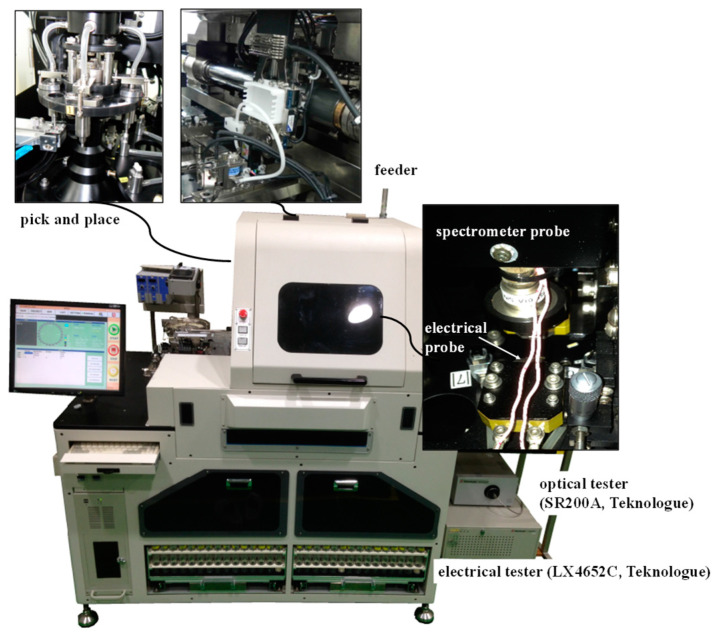
Automated LED inspection equipment for optical and electrical measurements.

As previously mentioned, this study aimed to transform the pre-existing expert system for quality control into a learning-based system that prioritizes scalability and reliability. Thus, we employed conventional measurement methods to gather learning data, ensuring seamless integration with the existing inspection line. An instrument for automated LED inspection was built as shown in Figure 1. Conventionally, both electrical and optical tests are conducted to check the quality of an LED package [29]. Commercialized electrical and optical testers (LX4652C, SR2000A, Teknologue, Kawasaki, Japan) were chosen and assembled into the system. The electrical tester was a voltage measurement device with precision current source control. In most cases, a tester with the μA–A range is sufficient for LEDs. A specified operational current rating is provided as a dominant specification because LEDs operate based on current, and the forward voltage is a resulting property as part of the product data [6]. Note that there have been attempts to use CCD images only [30], but spectrometers are usually required for reliable inspections.

**Figure 2:**
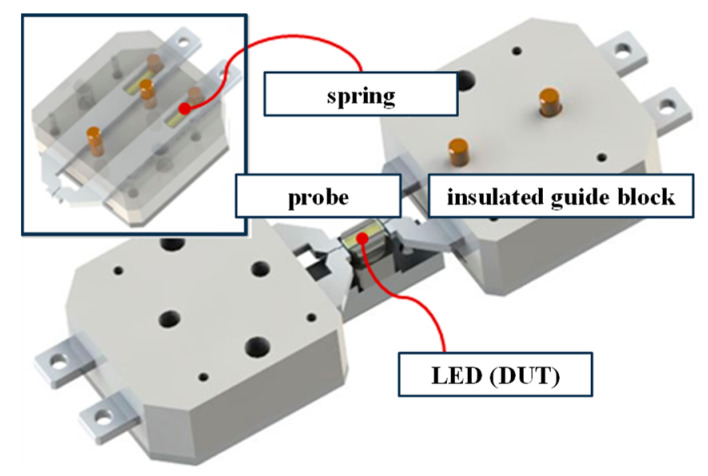
DUT handler with insulated block and probes.

In a mass production line, a pick-and-place device is required for inspection [31]. An automated pick-and-place device connected to a feeder was designed and implemented for automatically measuring the electrical and optical features. This work considered a white LED package with dual chips. This LED had only two electrodes exposed, allowing it to receive power for operation. Figure 2 shows the insulated jig that handles the DUT (device under test). The spring inside the jig applied the required force for contact between the LED electrodes and the test probes. With a durable palladium-coated electrical sensing probe, it was possible to quickly measure and record voltage in situations where current is controlled. As shown in Figure 2, the electrodes of the probe and LED were pressed together using spring force to measure the voltage under reliable conditions. Pneumatic control allowed for either contact or disconnection between the LED and probe electrodes. As mentioned earlier, the electrical tester used in this experiment could rapidly measure voltage under controlled current conditions, enabling voltage measurements across various current conditions.

Additionally, the optical characteristics of the LED package could be simultaneously measured during operation voltage application. Further details of the equipment are suppressed here. This sort of inspection system is quite widely used, but the classification algorithm was constructed manually based on the expertise of engineers. Conventionally, both electrical and optical data are used in LED inspection for quality control according to IEC 60081 [32].

### 2.2. Electrical Test

**Figure 3:**
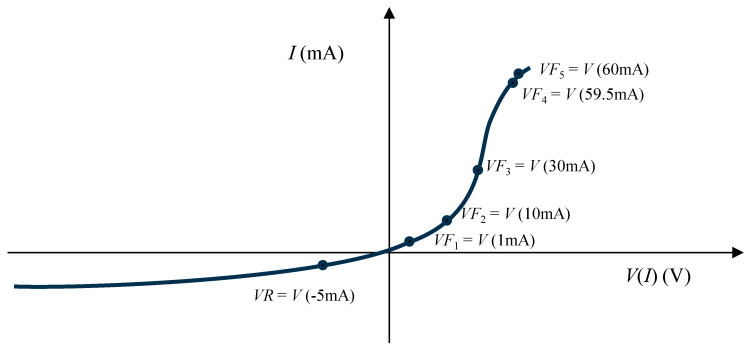
Voltage test under controlled current: *VR*, *VF*_1_, *VF*_2_, *VF*_3_, *VF*_4_ and *VF*_5_.

The current–voltage characteristic is the most fundamental and informative electrical characterization of an LED. The measured electrical data represents the device health, which is crucial for both development and manufacturing processes. During device development, the full *I–V* curve is measured by sweeping the current over the range of interest. This is required to understand the device’s behavior and performance [33]. For a single junction device, the current range can be very wide, spanning from tens to thousands of mA depending on the LED power, while the operating voltage ranges from about 1.5 to 4 V. The dynamic resistance is defined as the reciprocal of the slope of the *I–V* curve above the turn-on voltage. It is a measure of the bulk resistances of the semiconductors and metals in the LED device. In manufacturing, testing throughput remains a significant concern. To expedite testing, only specific predefined points on the *I*–*V* curve are measured. If these measured data points fall outside the specified range, the device is deemed defective. In this study, we measured voltages at six controlled currents, as illustrated in Figure 3. The details about the six measurement points are presented below.

A PN junction in an LED requires a certain turn-on (knee, threshold) voltage to begin conducting substantial current. The LED is operated at a forward voltage beyond the turn-on voltage while current flows through it. Typically, for standard silicon-based LEDs, the turn-on voltage is around 0.7 to 1.2 volts, reflecting interfacial properties in the LED device. Below the turn-on voltage, the LED behaves like an open circuit, and virtually no current flows. If an LED is defective, causing noticeable current flow below the turn-on (usually between 0.5 and 1.2 V), the LED is regarded as leaky [34]. The voltage at 1 mA denoted as *VF*_1_ in Figure 3 is used to judge the leakiness. On the reverse voltage side, little to no current may flow. LEDs are not purposely operated in this region (reverse bias) [31]. However, this reverse voltage can be used in testing as a metric of defectiveness. The negative voltage, *VR*, is measured at −5 mA as shown in Figure 3 [35]. In addition, four more forward voltages, *VF*_2_, *VF*_3_, *VF*_4_ and *VF*_5_, are measured at the currents indicated in Figure 3. The resistive region where an increase in voltage leads to a decrease in current increase rate is often observed in curves, and this is explained by the effect of an increase in resistance due to elevated temperature [36]. In this way, measuring the forward voltage for various current values has been proven useful for verifying the normality of the operating voltage. If the LED is functioning correctly, it is expected that *VF*_2_, *VF*_3_ and *VF*_4_ will be measured within a certain range, ideally reproducing its characteristic curve like Figure 3. Additionally, for a healthy LED, an increase in current beyond the operating voltage range does not significantly affect the forward voltage. Therefore, if significant variations are observed in *VF*_4_ and *VF*_5_, there is a higher likelihood that the LED is defective.

### 2.3. Optical Test

In this work, the peak wavelength *λ_p_* and the luminous intensity *Iv* were measured [37]. The peak wavelength signifies the wavelength at which an LED emits the greatest power [38]. It is determined from a chart depicting power density versus wavelength, where the peak wavelength corresponds to the point where the radiation energy in the spectrum is at its maximum. The popularity of the peak wavelength arises from its visually intuitive nature, making it easy to identify. Naturally, any alteration in this value can lead to variations in the characteristics of the LED.

**Figure 4:**
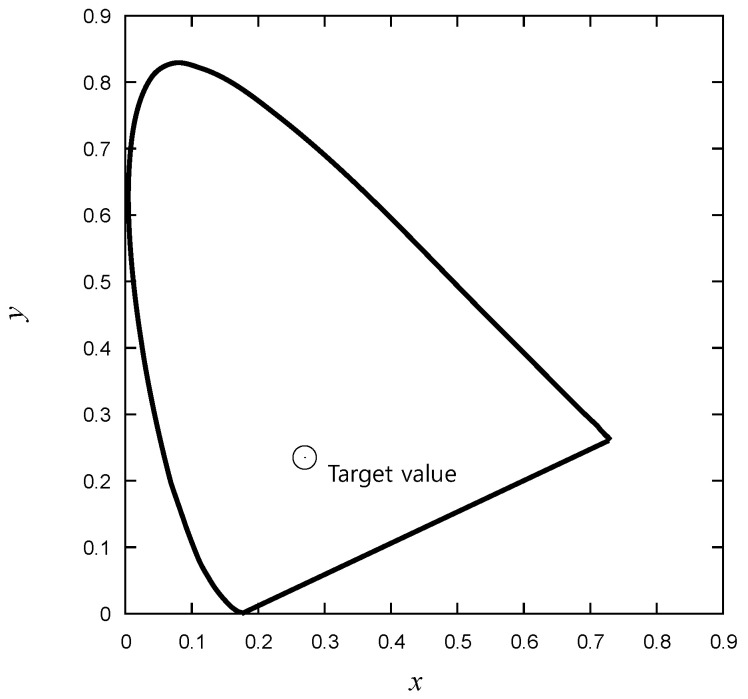
Measurement of *x* and *y* in CIE 1931 [6].

The luminous intensity is flux emitted integrated over a given directional range from the DUT and is conventionally expressed in Candela (Cd) [6,39]. Additionally, *x* and *y* values in the CIE 1931 diagram, as presented in Figure 4, were measured to capture the variation in colors. As a result, four optical measurement values were obtained, and when combined with the electrical test results for a single LED, a total of ten features were acquired.

### 2.4. Learning Data

**Figure 5:**
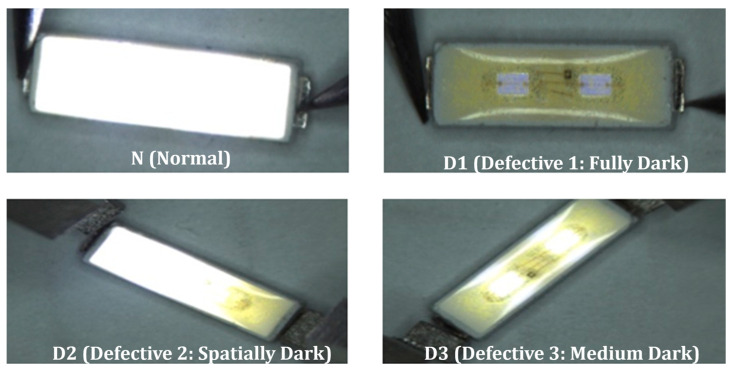
Defect classification to N, D1, D2 and D3 by visual observation of DUT.

Differentiating between satisfactory and faulty LED packages relies on the qualitative standards established for these components. In the context of crafting measurement devices for these components, only imperfections such as component breakage and insufficient luminosity were regarded as defects. This investigation encompassed three grades of defects, designated as D1, D2 and D3, as depicted in Figure 5. LED packages that do not respond to the current were categorized as D1. Partially luminous packages were classified as D2, while uniformly dim ones fell under the D3 category. Other LEDs were classified in the Normal category. The initial categorization was performed using a rough criterion based on *Iv*. Subsequently, multiple technicians conducted exhaustive examinations at least twice to finalize the categorization of both the training and validation data.

In the process of gathering training and validation data, the classification procedure was manually executed, with automated image acquisition with a CCD camera providing additional support. These four defect categories aligned with the classification outcomes to be predicted. Within this work, supervised learning was employed, utilizing the ten previously mentioned electrical and optical test features, to predict the specific category to which an LED belongs. A total of 8600 tests were conducted to collect data for both learning and validation purposes, with 6000 tests allocated for learning and 2600 for validation. While learning, *K*-fold cross-validation was conducted with *K =* 5. Note that the separate validation data were not used during *K*-fold cross-validation.

In the current investigation, the classification methods to be presented in the following sections were implemented by the ClassificationLearner and Deep Learning Toolbox in Matlab 2023a. The learning and classification were conducted on a Windows 11 machine with an x-64-based CPU (AMD Ryzen Threadripper PRO 3995WX 16 physical cores 3.9 GHz).

### 2.5. Discriminant Analysis

In order to check if the tested LEDs can be classified by a simple statistical method, discriminant analysis (DA) was employed. Discriminant analysis has been adopted in classification processes for manufacturing. The method is renowned for classification of the new observations based on the prebuilt predictor variables. Several studies have utilized LDA (linear discriminant analysis) and QDA (quadratic discriminant analysis) for the purpose of categorizing defects based on data collected during the manufacturing process. [18,40,41,42].

Let us describe the linear DA (LDA) briefly. Here, these variables are called the *k*-th canonical variables, which are [43,44]

(1) $$V_{k}\left(x\right)=u_{k0}+\sum_{i=1}^{q}u_{ik}x_{i}\;\mathrm{for}\;k=1,\cdots ,N$$

where ***x*** is an observation vector with *q* members for a test. In this work, *q* = 10, and ***x*** is defined as

(2) $$x=\left[x_{1},\cdots ,x_{q}\right]=\left[VR,\cdots ,\lambda_{p}\right]$$

The coefficients *u*_ik_ in Equation (1) are set to find the linear combination of the *q* variables that maximizes the ratio of between-group to within-group variation. The formed canonical variates can then be used to discriminate between groups. In LDA, the number of the canonical variables is usually set as one smaller than the number of classes, *N*. As there are four classes shown in Figure 5, three variables, *V*_1_, *V*_2_ and *V*_3_, were evaluated during the LDA. This work also performed QDA, but the details are suppressed here [44].

### 2.6. Neural Network

**Figure 6:**
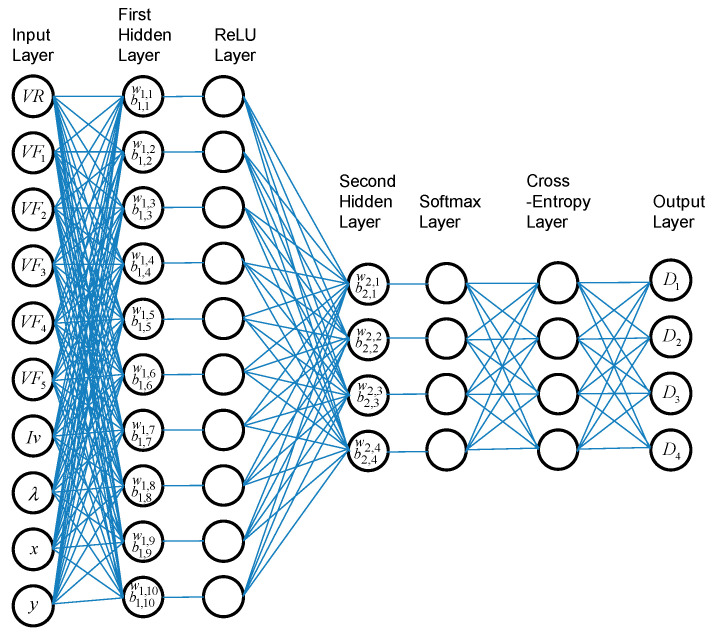
The classification neural network scheme employed in this work.

Neural networks for classification are a type of machine learning model that can be trained to categorize input data into different classes. In this context, the network learns to establish a relationship between input features and class labels, the NN structure in this work. A common architectural setup includes using the rectified linear unit (ReLU) as the activation function to mitigate the vanishing gradient problem [45] and employing Softmax at the output layer to transform the outputs into meaningful probability values [46]. Figure 6 shows a typical shallow NN for classification, which was employed in this work [47].

Furthermore, in the final classification layer, the cross-entropy method was employed. Cross-entropy is a widely used loss function for classification tasks. It quantifies the dissimilarity between the predicted probability distribution of a class and the actual probability distribution of that class. When training a classification model, the objective is to minimize the cross-entropy loss, signifying that the model should learn to predict the correct class label with the highest probability. If the value of the *j*-th neuron is *Y_j_*, and its corresponding target value is *T_j_*, the loss function is expressed by the following equation [48]:

(3) $$L=-\sum_{j=1}^{K}\;\;T_{j}\ln Y_{j}+\left(1-T_{j}\right)\ln\left(1-Y_{j}\right)$$

Here, we present the classification results when *L* is minimized.

### 2.7. Decision Tree

A decision tree is a supervised learning algorithm primarily utilized in classification tasks [49]. It involves a predefined target variable and is tailored for solving such problems. Especially in manufacturing processes, DTs are particularly well suited for classification tasks as they are purposefully designed for this specific purpose [50,51,52,53,54]. The data need to be labeled with a single categorical response variable, which corresponds to a process result. As previously described, each LED examined in this study must be assigned one of the categories: D1, D2, D3 or Normal. Leaf nodes correspond to potential values of the response variable, while non-leaf nodes correspond to a condition by a predictor feature. Each feature node divides a set of instances into subsets with the root node encompassing all instances. The key question is how this identification process takes place, including variable selection and partitioning. Decision trees employ various algorithms to accomplish this task.

During tree construction, the primary aim is to minimize the complexity, or impurity, at each node. In essence, the goal is to create a tree with a low impurity index, which quantifies the mixing degree within the leaf (result) node. Three common impurity indices are utilized: entropy, the Gini diversity index and the information gain ratio. The Gini diversity index is frequently favored due to its sensitivity to misclassification, computational efficiency and robustness in response to outliers. This work employed the following Gini index to check the impurity [55]:

(4) $$G\left(p\right)=1-\sum_{k=1}^{K}p_{k}$$

Here, *p_k_* is the probability of being classified into the *k*-th class. In this work, a binary tree was constructed by splitting a particular observation based on the choice that minimizes the aforementioned value at each node of the tree by following the standard CART algorithm [56,57]. Through the repetition of this process, a regression tree that is well fitted to the training data could ultimately be generated. At a specific node, the split was performed through the application of a binary judgment to a single measurement variable. Once the tree was fully built, if new data were input, the classification response was obtained by following the tree until the leaf node was reached.

The current configuration employed a maximum limit of 100 splits. This research endeavor additionally sought to ascertain the feasibility of diminishing the quantity of measurement variables by conducting the classification task solely based on either optical or electrical data. The rationale behind this pursuit lies in the recognition that measurements invariably entail associated costs, and a reduction in the quantity of measurements holds the potential to yield cost savings within the domain of quality control.

## 3. Results

### 3.1. Observation of Learning Data

In conventional quality control frameworks, human-derived rule-based expert systems are prevalent. However, they struggle with scalability, timely updates, and achieving full automation. At times, the data may inherently exhibit patterns that suggest rules, which can streamline the construction of an expert system. Thus, to assess the viability of a learning-based model, it is essential to analyze the correlations within the gathered data. A comprehensive analysis is not required since specific subsets of data will be enough to describe the characteristics of the data.

**Figure 7:**
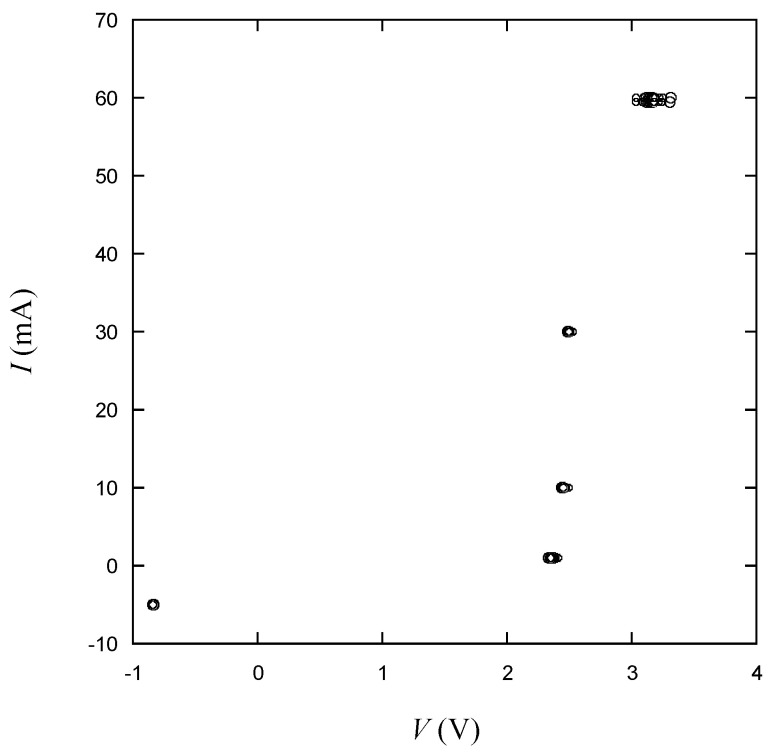
Current–voltage data for 15 normal samples.

First, the electrical measurement data will be examined. As plotted in Figure 7, a notable increase in current is evident among *VF*_1_, *VF*_2_ and *VF*_3_, accompanied by a slight rise in voltage difference. For *VF*_4_, it is observed that the voltage exhibits a relatively substantial increase in comparison to *VF*_3_. *VF*_4_ and *VF_5_* are difficult to distinguish on the plot due to the low current difference. In general, a resemblance to the pattern illustrated in Figure 3 is observed.

**Figure 8:**
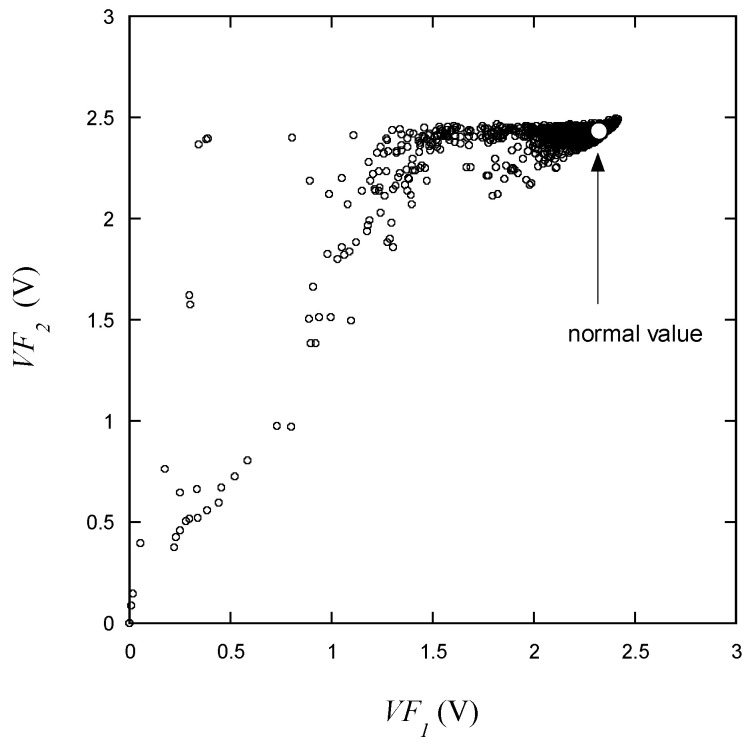
*VF*_2_ vs. *VF*_1_ for the learning data.

In Figure 8, *VF*_2_ is plotted relative to *VF*_1_. In the range measured with low voltage, it appears that there is a correlation between the two voltages. However, near the normal operating range, *VF*_2_ maintains a consistent voltage even as *VF*_1_ varies. Within the normal operating range, *VF*_1_ is observed near its maximum value, while *VF*_2_ is predominantly clustered around the observed maximum voltage. The normal value in the figure designates the target operational point of the device.

**Figure 9:**
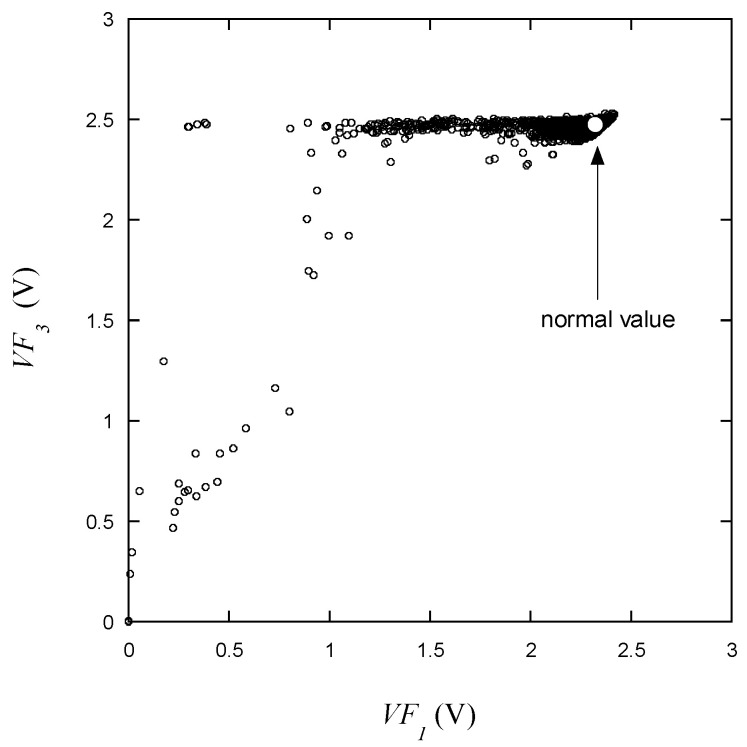
*VF*_3_ vs. *VF*_1_ for the learning data.

**Figure 10:**
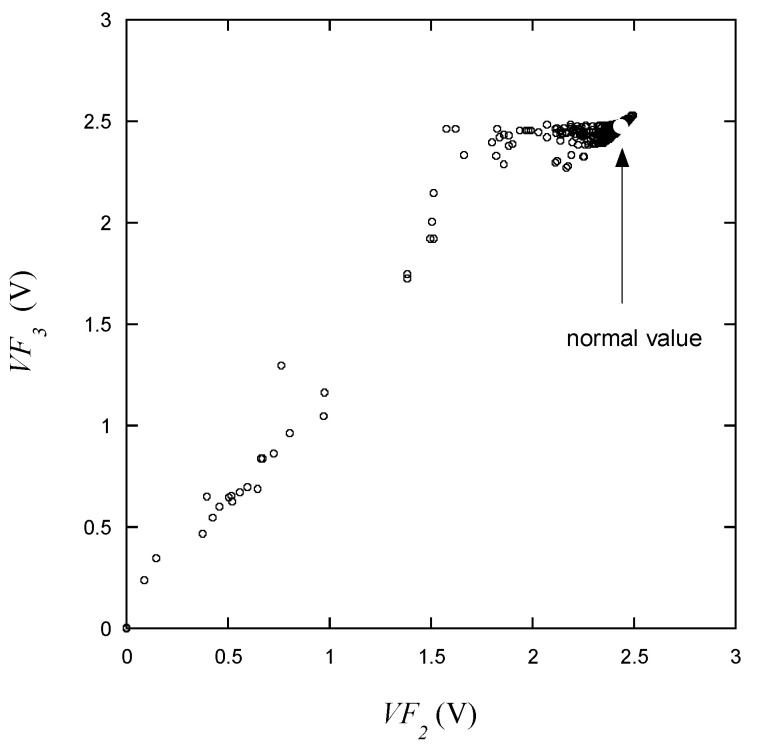
*VF*_3_ vs. *VF*_2_ for the learning data.

The variation in *VF*_3_ with respect to *VF*_1_ as presented in Figure 9 is similar to what is depicted in Figure 8. From this, it can be predicted that the behaviors of *VF*_3_ and *VF*_2_ will be generally alike. This is indicative of the fact that *VF*_1_ is measured at low current, thus reflecting a substantial variation in behavior at low currents in accordance with the manufacturing characteristics of the LED. As can be seen in Figure 10, as mentioned earlier, *VF*_3_ and *VF*_2_ exhibit a strong correlation at low voltages. Furthermore, it can also be observed that *VF*_3_ maintains a relatively consistent behavior near the maximum voltage during normal operation. 

**Figure 11:**
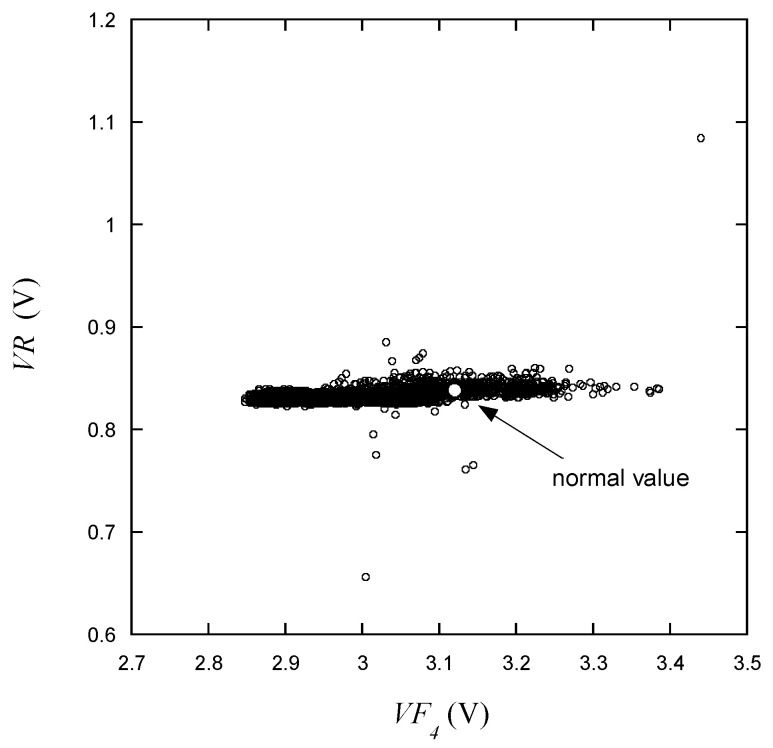
*VR* vs. *VF*_4_ for the learning data.

In Figure 11, the variation in *VR* with respect to *VF*_4_ is shown. It can be observed that *VR* remains relatively constant while *VF*_4_ varies over a wide range of voltages. From these characteristics, it can be predicted that *VR* is likely to be considered defective if it deviates from the narrow range indicated, whereas *VF*_4_ may need to be assessed over a wider range.

**Figure 12:**
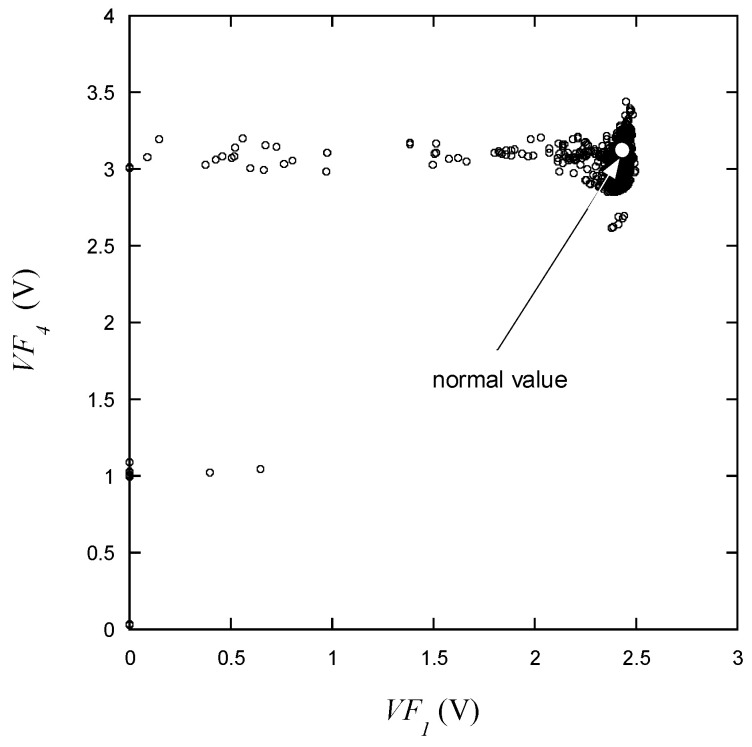
*VF*_4_ vs. *VF*_1_ for the learning data.

**Figure 13:**
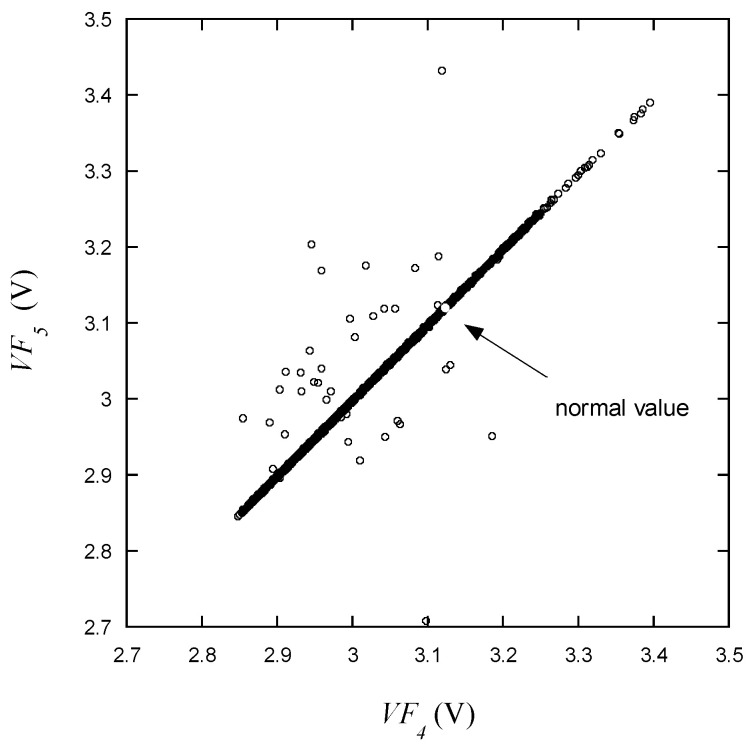
*VF*_5_ vs. *VF*_4_ for the learning data.

On the other hand, as mentioned earlier, *VF*_1_ exhibits a wider range of variation than *VF*_4_. It is particularly noteworthy that voltages significantly deviating from the normal range can be observed in Figure 12. Values lower than the normal voltage are mostly presumed to be related to leakiness. It can be predicted that *VF*_4_ and *VF*_5_, which are measured within very close current ranges, will be strongly correlated with each other. As presented in Figure 13, *VF*_5_ and *VF*_4_ exhibit a highly linear relationship, and data points deviating from this correlation are expected to have a high likelihood of being defective. Intuitively classifying LEDs based solely on the distribution of voltage data appears to be quite challenging. Given the significant variability in voltage measurement values, it is predicted that the criteria will vary from *VR* to *VF*_5_. For instance, if *VF*_1_ is measured at a very low level, there is a high likelihood that *VF*_3_ is also in the abnormal range, but even if *VF*_3_ is within the normal range, *VF*_1_ is likely to be in the abnormal range.

**Figure 14:**
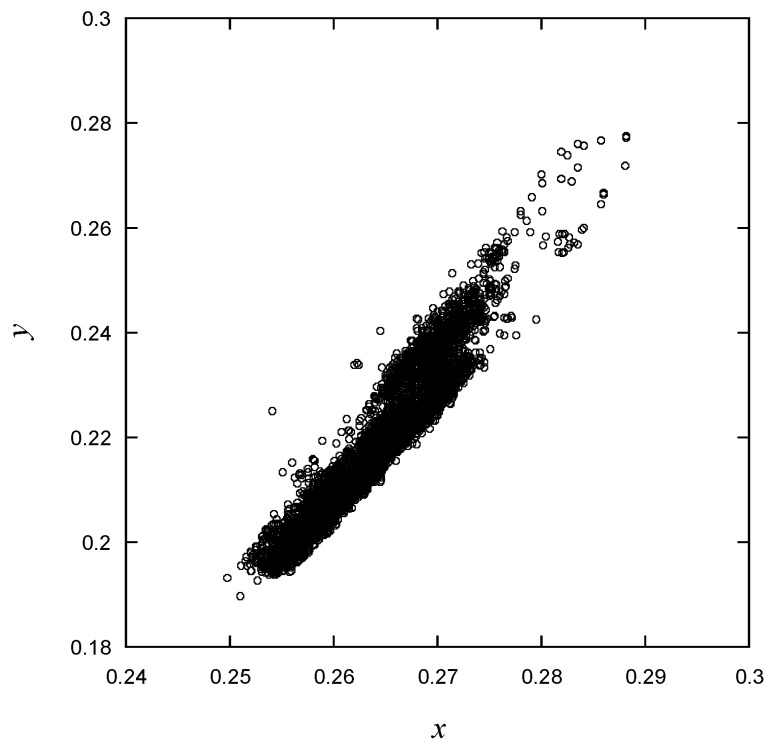
y vs. x in CIE1931 for the learning data.

**Figure 15:**
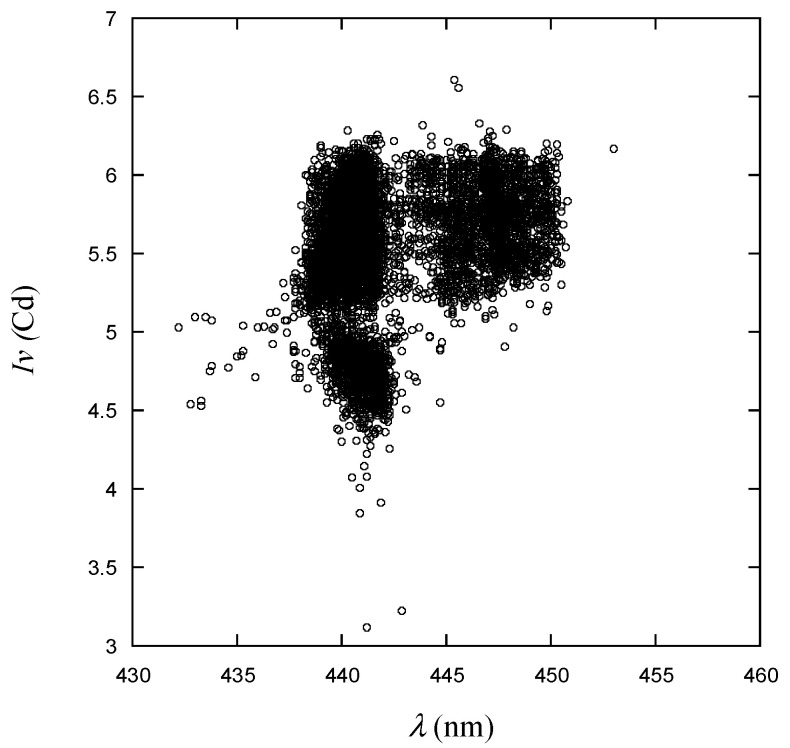
Luminous intensity vs. wavelength for the learning data.

As can be observed in Figure 14, the *x* and *y* CIE1931 values exhibit a strong linear correlation with each other. Many data points are densely clustered near the target values presented in Figure 4. The data to be used for training are presented in Figure 15, showing luminous intensity as a function of wavelength. On the graph, the data points are divided into three distinct groups. While a specific group may not necessarily signify good or defective products, it is evident that there are distinct differences between them. Particularly, products with different *Iv* values can be classified into different grades, but this study does not consider grading for defect-free products.

**Figure 16:**
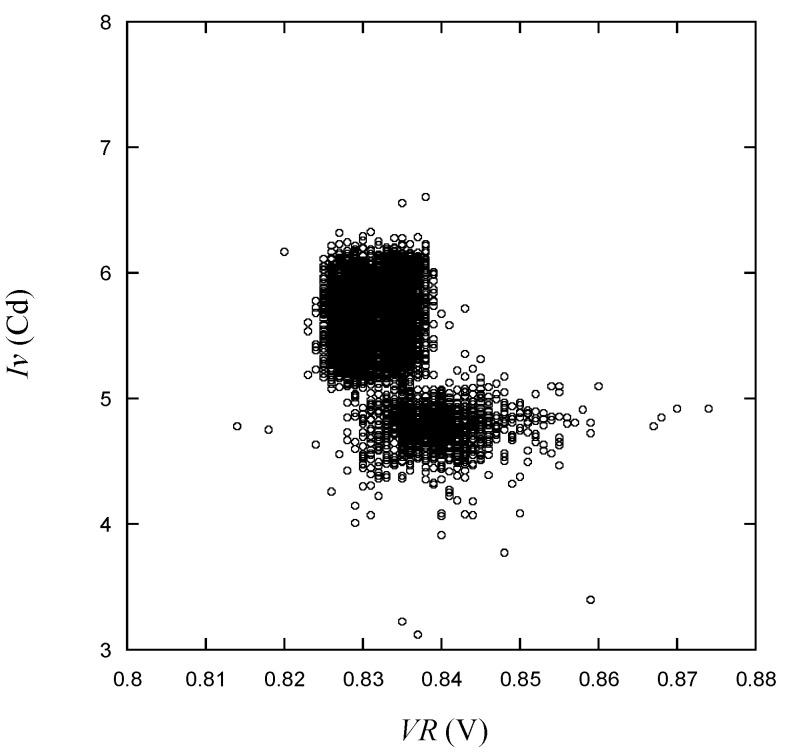
*Iv* vs. *VR* for the learning data.

**Figure 17:**
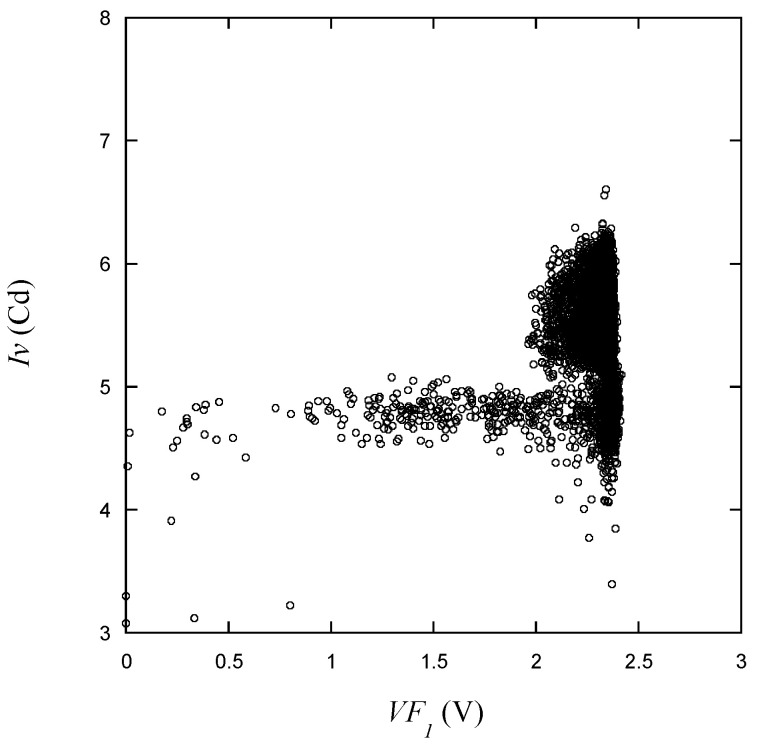
Luminous intensity vs. *VF*_1_ for the learning data.

Luminous intensity, as seen in Figure 16, varies according to *VR*, with two distinct groups forming and values outside the range of the scattered group being visible in the vicinity. In Figure 17, the relationship between *Iv* and *VF*_1_ is plotted. When *VF*_1_ deviates from the normal range and records a low voltage, there is a tendency for *Iv* values to generally appear in the lower group. In cases where *Iv* values are high, *VF*_1_ is typically observed to be close to the normal voltage range. Taken together with the observations regarding voltage and optical measurements presented earlier, it appears to be quite challenging to establish quality criteria solely based on the values observed in the plots.

Setting criteria for an expert system from such data may be somehow achievable for engineers with a high level of understanding of the both electrical and optical characteristics of LEDs, coupled with deep insight and experience, but it is expected to be a time-consuming process to ensure reliability. As evident from the results presented earlier, it seems difficult to express the relationships between the data as simple functions. Moreover, if quality judgments are made based on the range of each feature, many defect-free products may be erroneously rejected, which, as mentioned earlier, could significantly reduce yield. Therefore, it appears necessary to establish a quality control process through automated methodologies such as DA, NNs and DTs.

### 3.2. Discriminant Analysis

**Figure 18:**
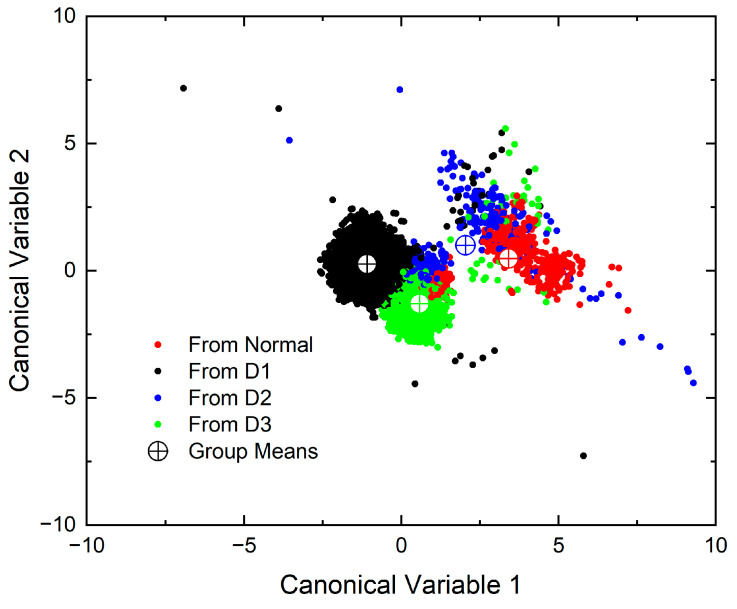
Canonical variable 2 alongside canonical variable 1 from the discriminant analysis.

**Figure 19:**
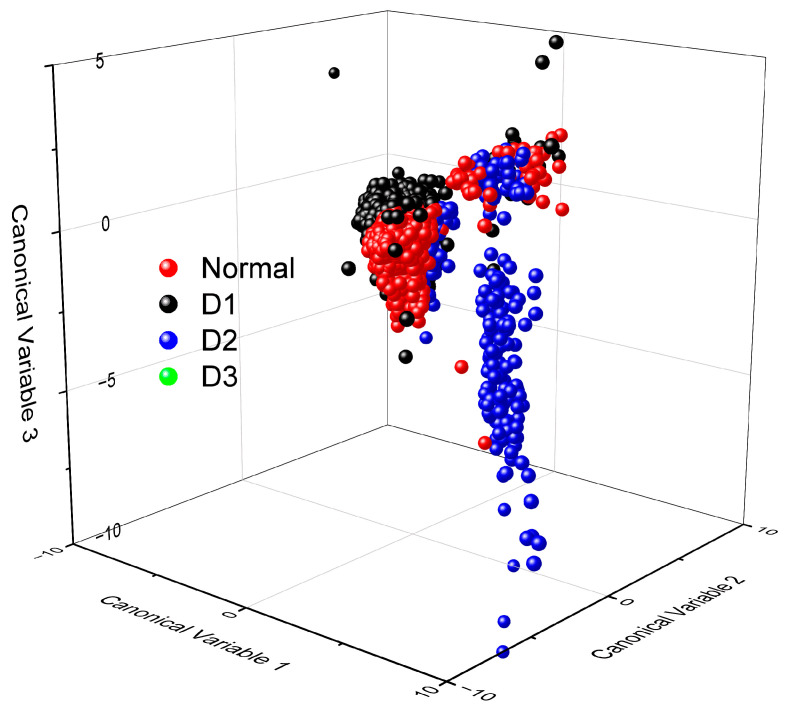
Canonical variable 3 alongside canonical variables 1 and 2 from the discriminant analysis.

The results of discriminant analysis are presented in Table 1. For each learning dataset, the coefficients from Table 1 were substituted into Equation (1), and canonical variables 1 and 2 were plotted using the measured values, as depicted in Figure 18. It is evident that the displayed points form distinct groups. However, even variables belonging to the Normal group are observed to be widely scattered, and points from the D1, D2 and D3 groups are also highly dispersed. Figure 19 represents the data in three-dimensional space for the three canonical variables. The Normal group clearly divides into two distinct groups, and the points corresponding to D2 are widely spread, indicating the difficulty in discrimination using linear discriminant analysis (LDA).

**Figure 20:**
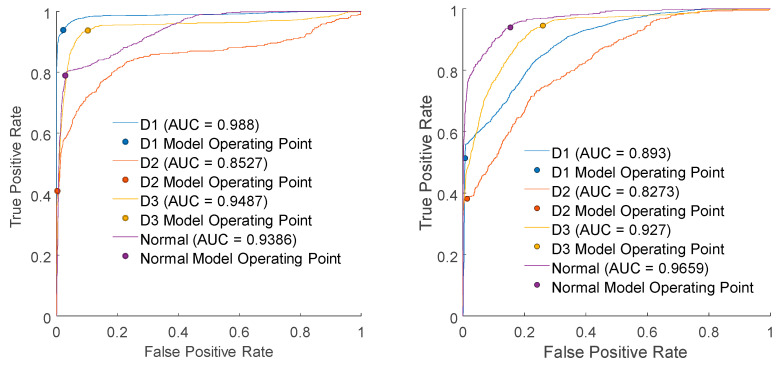
Receiver operating characteristics from LDA (**left**) and QDA (**right**).

The evaluation results using validation data are presented in Table 2, where the discriminative success rate for the Normal category reaches only 77.92%. As evident from Figure 19 and Figure 20, it can be inferred that the distribution of data may pose challenges for successful classification based solely on variance. The ROC (receiver operating characteristic) evaluates the trade-off between the true positive rate (TPR) and the false positive rate (FPR). The ROC curve shown in Figure 20 illustrates that there is some discernible resolving power; however, it falls short of meeting the requirements for an effective quality control process.

When QDA was employed, markedly different results were observed. A distinct improvement was not seen in the Normal category, while a 39% reduction in prediction accuracy for D1 was observed, as can be observed in Table 3. This dramatic difference may have been caused by the complex distribution of data in both the Normal and D1 categories. Notably, the presence of numerous outliers in the D1 category would have rendered the prediction task quite challenging. A previous study on turning process analysis reported an accuracy of 86.9% when employing QDA [18]. However, it is important to note that the features used in this study may not be well suited for discriminant analysis (DA). Nevertheless, it is essential to acknowledge that DA provides valuable insights into data distribution, even if it may have limited discriminatory power.

### 3.3. Neural Network

**Figure 21:**
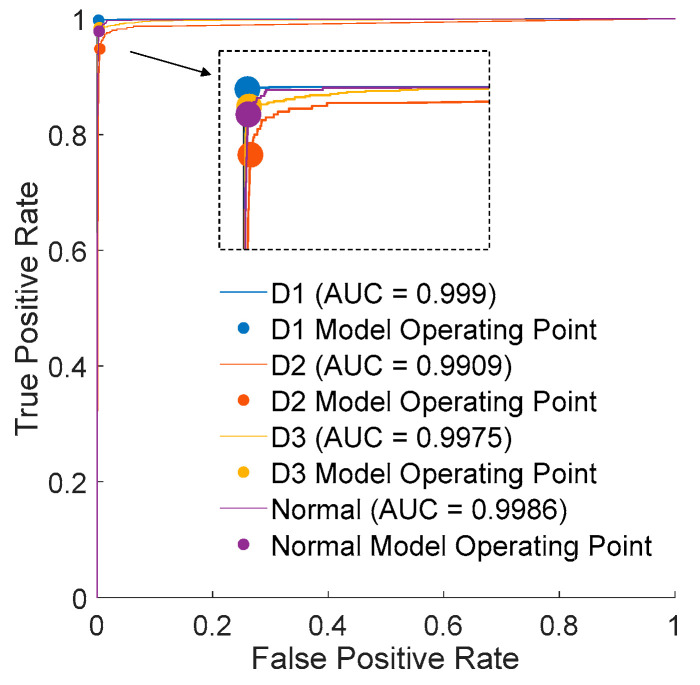
Receiver operating characteristics from neural network.

Multiple configurations of NNs with additional layers and neurons have been tested, yet the arrangement presented in Figure 6 has emerged as the most effective for this dataset. The learning process has been conducted with the training dataset. The outcomes of an evaluation with 5-fold cross-validation were found to be 97.4% true for normal data (see Figure S1 in the supplemental data). The results using the validation data are presented in Table 4. The accuracy in correctly classifying normal data reached 97.8%, signifying a substantial improvement when compared to the results obtained using LDA. The ROC curve in Figure 21 shows very high operating points with AUC over 0.99.

Nevertheless, this level of accuracy for the category Normal still falls short of meeting the stringent requirements for effective deployment within quality control of LEDs. In addition to the setup shown in Figure 6, various NN designs have been tried. In previous works, there have been successful applications of deep NN in classification or detection [19,58,59,60,61]. Intriguingly, augmenting the quantity of fully connected layers in this work led to a decrement in the recognition rate, and various combinations failed to yield discernibly improved outcomes. This might be featured to overfitting [62], data deficiency or vanishing gradients [63], which are persistent issues in deep learning [64]. Given the limited batch size of the current data, applying convolution becomes challenging, making it difficult to enhance performance of the NN further.

### 3.4. Decision Trees

**Figure 22:**
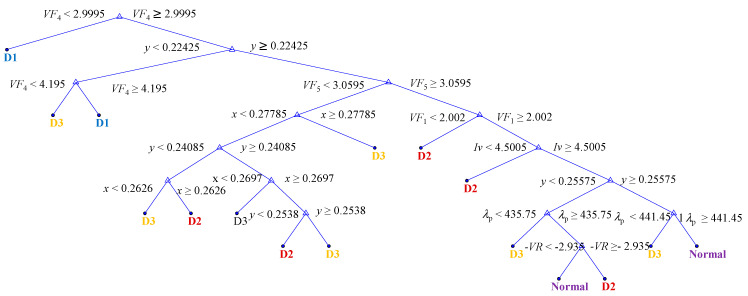
The built decision tree with both optical and electrical measurements.

**Figure 23:**
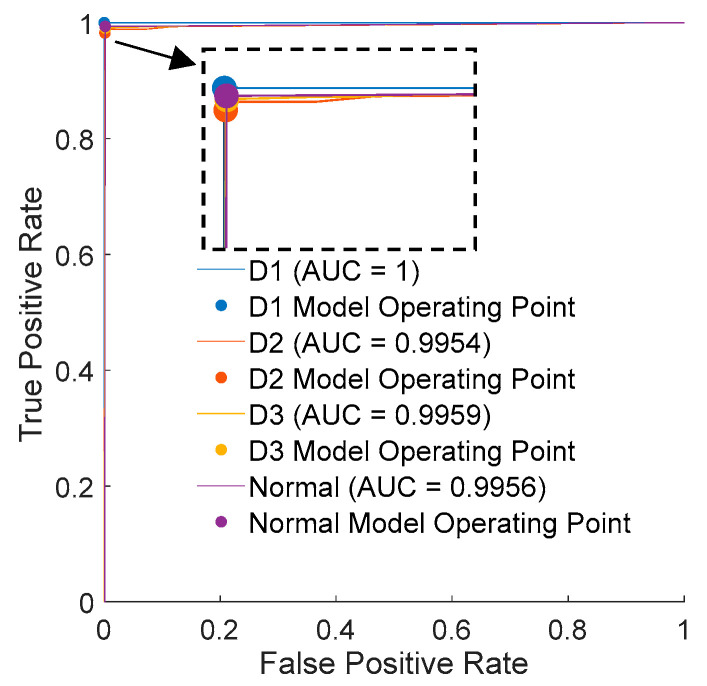
Receiver operating characteristics from the decision tree.

The results of the training process using the training data have culminated in the formation of the DT illustrated in Figure 22. While the tree is not exceedingly simplistic, it achieves classification with a maximum of 14 splits. The evaluation by 5-fold cross-validation reached 98.9% true for normal data (see Figure S2 in the supplemental data). Additionally, the evaluation results using validation data are presented in Table 5. These results demonstrate that 99.4% of Normal LEDs were correctly classified as Normal, and D1 achieved a perfect 100% classification rate. However, D2 exhibited a 1.7% misclassification rate. The ROC curve presented in Figure 23 exhibits an unusual characteristic in machine learning, with a shape that is notably close to TPR = 1, which is a rare occurrence. As a result, the obtained AUC (area under the curve) value is 0.9956 for the Normal category.

The DT shown in Figure 22 is relatively straightforward to implement on a computer and exhibits a high success rate in classification. Consequently, it is anticipated that it can be employed for quality management purposes. In light of these findings, it is believed that the construction of a DT based on the training process can enable the implementation of a classification system logic without the need for expert assistance. Let us conduct a brief analysis of the resulting tree. In the initial and second splits, the tree identifies all instances of D1. As anticipated, there may be some ambiguity between D2, D3 and Normal. As we traverse down the tree, D2 and D3 are successfully classified. Ultimately, any remaining instances of DUT that do not fall into the D2 or D3 categories are labeled as Normal.

The DTs obtained using only electrical measurements and optical measurements separately, from the same dataset, yielded TPRs of only 77.1% and 79.6% for Normal data, respectively (see Tables S1 and S2 in the supplemental data). Furthermore, the structures of the trees were exceedingly complex, with 79 and 74 splits (see Figures S3 and S5 in the supplemental data). Therefore, it can be concluded that training should involve the simultaneous utilization of both electrical and optical measurements. An earlier study has also indicated that a DT method based on random forests outperforms NNs [65].

As shown in Figure 9, Figure 10, Figure 11, Figure 12, Figure 13 and Figure 14, the decision boundaries are nonlinear. However, decision trees are capable of capturing complex nonlinear relationships in data through a series of simple decision rules. In some cases, this simplicity can lead to a more efficient representation of the underlying data structure compared to NNs. As aforementioned, the DT is simpler and more interpretable. Moreover, it is robust in response to outliers as it can split the data into distinct regions.

### 3.5. Computational Aspect

On the specified machine, the training times for QDA, NN and DT are 8.1 s, 18.2 s and 8.2 s, respectively. Meanwhile, the prediction speeds are 100,000 samples per second, 150,000 samples per second and 120,000 samples per second, respectively. During validation, the total costs have been evaluated as 3366, 108 and 18, respectively. The total cost is calculated as the weighted sum of false negatives and false positives. Additionally, the model sizes are 16 kB, 9 kB and 11 kB, respectively. In addition to the excellence of the DT in accuracy, it is well rounded in terms of computational efficiency.

## 4. Conclusions

In this study, a supervised learning method was employed to enhance the logic for classifying mass-produced LEDs. The hardware system responsible for conducting the tests was described, along with the presentation of the test performance method. The test results were categorized into three defective categories and one normal category, and learning was carried out using 6000 training data points and 2600 validation data points. Six current-dependent voltage measurements and four optical measurement values served as features for each data point.

Upon evaluating the learning performance, it was found that linear discriminant analysis achieved a true positive rate of only 77.9% for normal products, indicating insufficient suitability for quality control. Following supervised learning using a neural network, a substantial improvement in the true positive rate to 97.8% was achieved. However, the 2.2% false negative rate was considered significant in product classification. To address this concern, learning was undertaken to construct a binary decision tree, resulting in highly reliable outcomes with a true positive rate of 99.4% from just 14 splits, underscoring its effectiveness in product classification.

## Figures and Tables

**Table 1:** Canonical coefficients of the discriminant analysis.

u	Canonical Variable 1	Canonical Variable 2	Canonical Variable 3
Constant	−19.30198	16.4686	1.5625
VR	1.44599	−0.93851	1.5458
VF_1_	0.51609	−1.2343	8.01005
VF_2_	2.69957	−4.0174	−2.38663
VF_3_	−6.56315	8.56547	−1.57562
VF_4_	7.84453	−7.20327	−1.99377
VF_5_	5.58177	−3.22984	0.14349
Iv	1.3556	−135.89407	−114.01634
x	45.32186	135.81131	67.99315
y	−1.26239	−0.51891	−0.99715
λ_p_	−0.03245	0.03286	0.03923

**Table 2:** Prediction by LDA using the validation data (*TPR).

		Predicted Group			
		D1	D2	D3	Normal
Validation data	D1	91.0% *	0.9%	7.1%	0.9%
	D2	4.7%	41.3% *	35.9%	18.1%
	D3	1.4%	1.0%	93.4% *	4.1%
	Normal	0.0%	4.5%	17.5%	77.9% *

**Table 3:** Prediction by QDA using the validation data (*TPR).

		Predicted Group			
		D1	D2	D3	Normal
Validation data	D1	52.0% *	1.9%	29.2%	17.0%
	D2	0.7%	38.2% *	27.8%	33.4%
	D3	1.1%	0.2%	94.5% *	4.1%
	Normal	0.2%	0.8%	5.1%	94.0% *

**Table 4:** Prediction by neural network using the validation data (*TPR).

		Predicted Group			
		D1	D2	D3	Normal
Validation data	D1	99.8% *	0.1%	0.1%	0.0%
	D2	0.4%	94.8% *	2.0%	2.8%
	D3	0.3%	0.9%	98.4% *	0.3%
	Normal	0.0%	1.5%	0.6%	97.8% *

**Table 5:** Prediction by decision tree using the validation data (*TPR).

		Predicted Group			
		D1	D2	D3	Normal
Validation data	D1	100.0% *	0.0%	0.0%	0.0%
	D2	0.0%	98.3% *	1.3%	0.4%
	D3	0.0%	0.4%	99.1% *	0.5%
	Normal	0.0%	0.2%	0.5%	99.4% *

## Data Availability

The datasets generated and supporting the findings of this article are obtainable from the corresponding author upon reasonable request.

## Supplementary Materials

The following supporting information can be downloaded at: https://www.mdpi.com/article/10.3390/mi15040457/s1. Figure S1: Validation confusion matrix for neural network by 5-fold cross-validation; Figure S2: Validation confusion matrix for decision tree by 5-fold cross-validation; Figure S3: Built decision tree only with electrical measurements; Figure S4: Classification results using the decision tree only with electrical measurements; Figure S5: Built decision tree only with optical measurements; Figure S6: Classification results using the decision tree only with optical measurements; Table S1: Prediction by decision tree only using the electrical data; Table S2: Prediction by decision tree only using the optical data.
