# Supplementary material for: Classification of LED Packages for Quality Control by Discriminant Analysis, Neural Network and Decision Tree
Source: Micromachines (Basel). 2024 Mar 28;15(4):457. doi: 10.3390/mi15040457 (PMC11051947; doi:10.3390/mi15040457)
Supplement: Supplementary file 1 [file micromachines-15-00457-s001.zip › micromachines-2830777-supplementary.pdf]

Supplemental Data

Classification of LED packages for quality control by discriminant analysis, neural network and decision tree

Shim and Kim

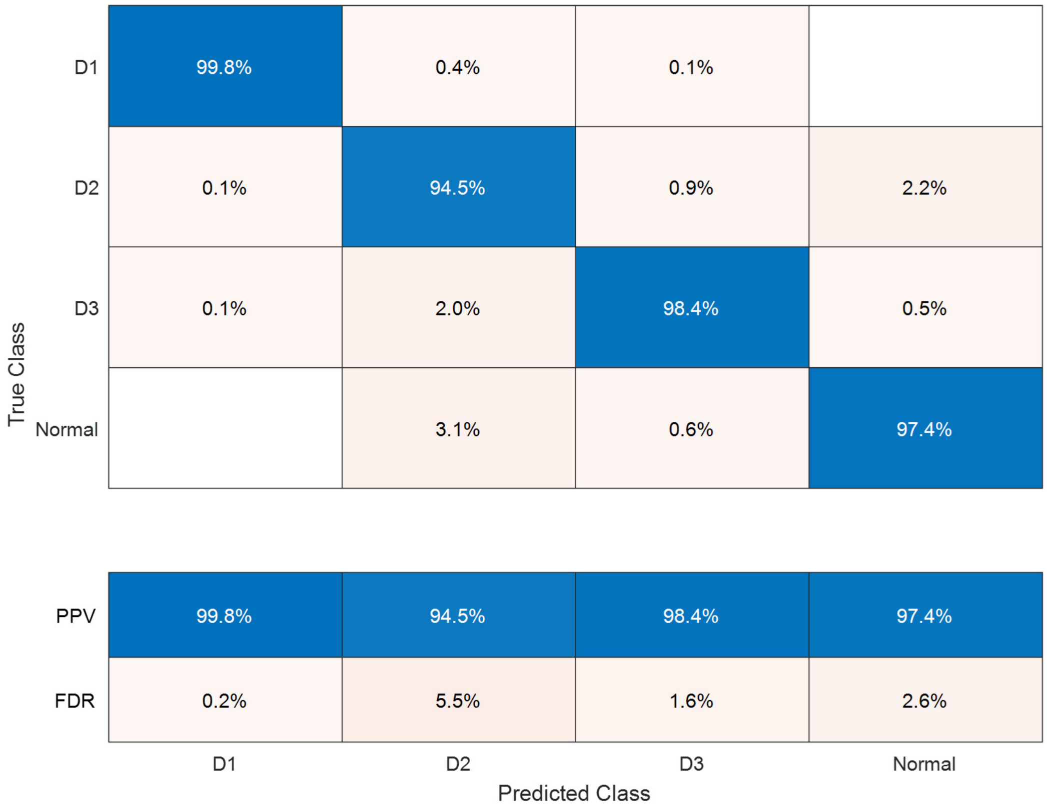

Figure S1. Validation confusion matrix for neural network by 5-fold cross-validation.

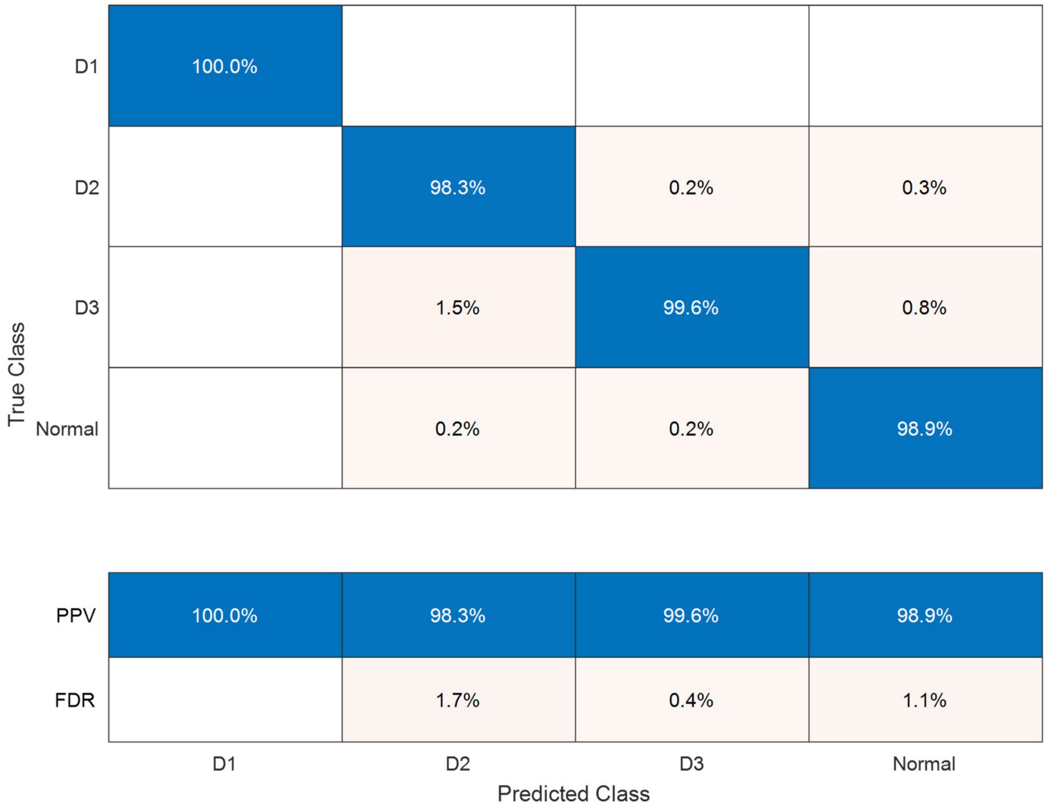

Figure S2. Validation confusion matrix for decision tree by 5-fold cross-validation.

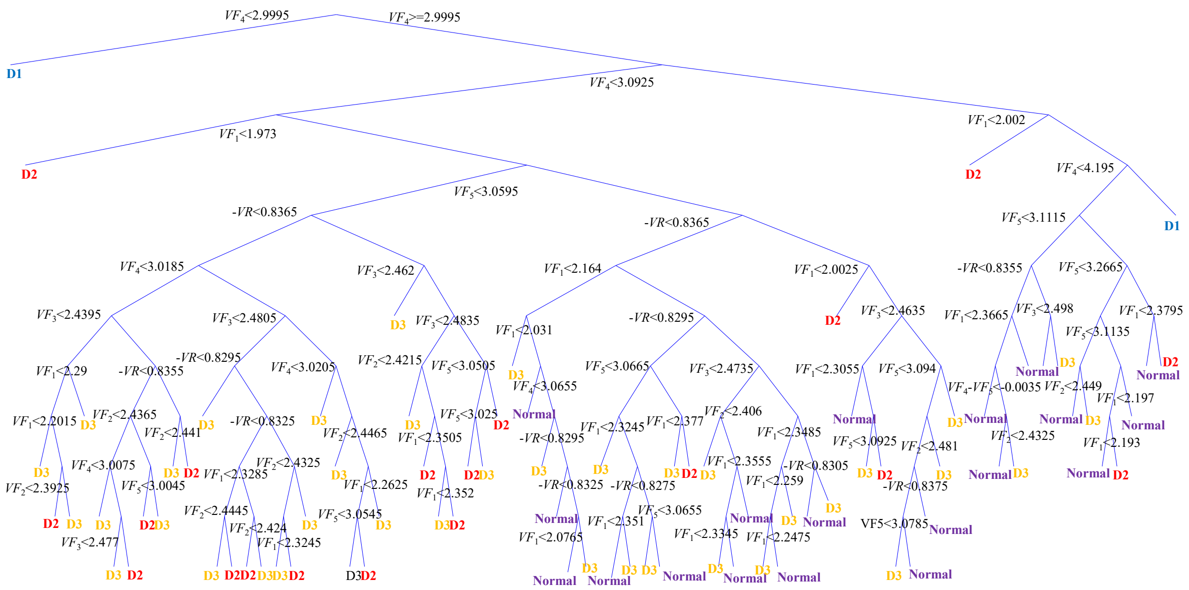

Figure S3. Built decision tree only with electrical measurements.

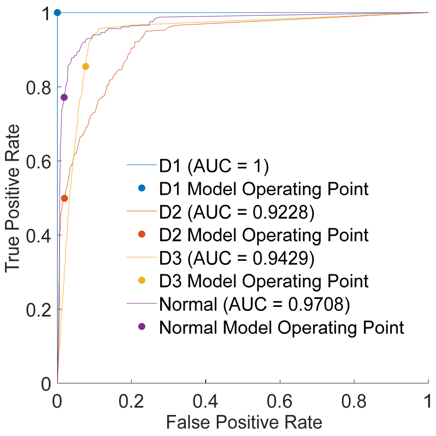

Figure S4. Classification results using the decision tree only with electrical measurements.

Table S1. Prediction by decision tree only using the electrical data.

		Predicted Group			
		D1	D2	D3	Normal
Validation data	D1	100.0%*	0.0%	0.0%	0.0%
	D2	0.0%	49.9%*	47.5%	2.6%
	D3	0.0%	7.8%	85.5%*	6.7%
	Normal	0.0%	1.1%	21.8%	77.1%*

*TPR

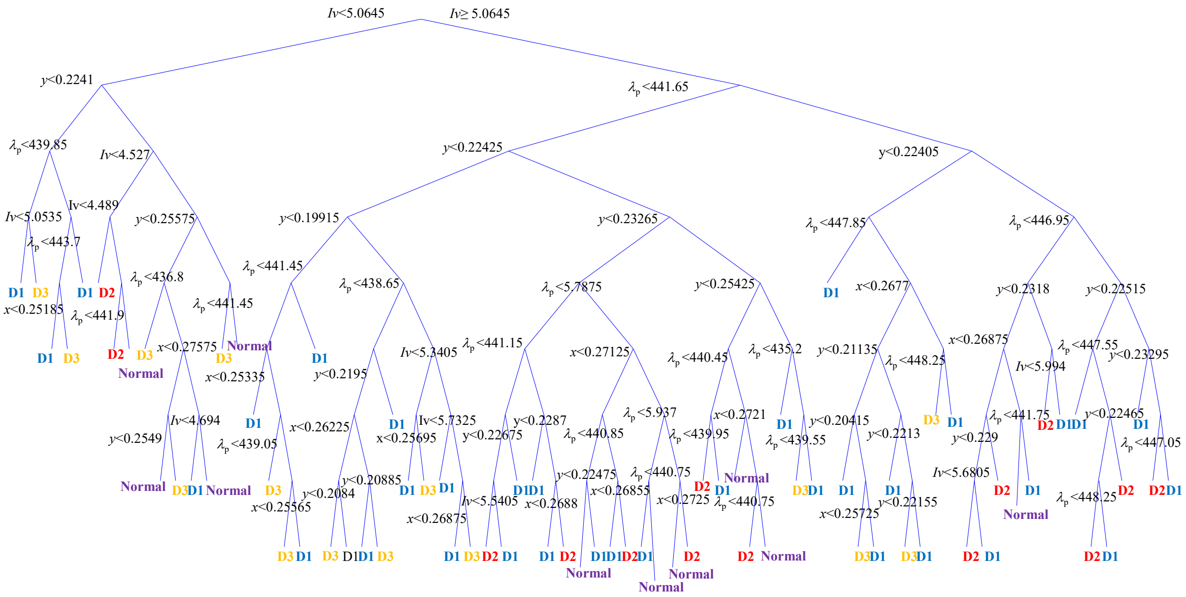

Figure S5. Built decision tree only with optical measurements.

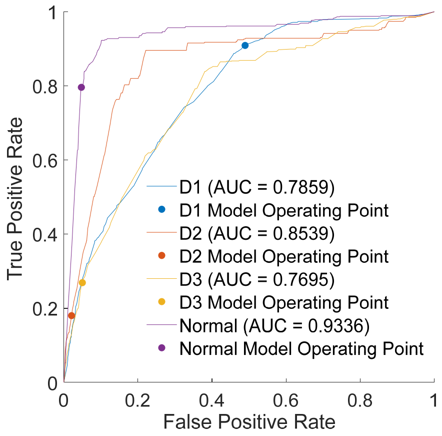

Figure S6. Classification results using the decision tree only with optical measurements.

Table S2. Prediction by decision tree only using the optical data.

		Predicted Group			
		D1	D2	D3	Normal
Validation data	D1	90.9%*	1.9%	6.3%	1.0%
	D2	35.8%	18.0%*	0.2%	46.0%
	D3	72.6%	0.1%	26.7%*	0.5%
	Normal	11.9%	7.4%	1.1%	79.6%*

*TPR
